# Prematurity is a critical risk factor for respiratory failure after early inguinal hernia repair under general anesthesia

**DOI:** 10.3389/fped.2022.843900

**Published:** 2022-07-25

**Authors:** Sebastian Schroepf, Paulina M. Mayle, Matthias Kurz, Julius Z. Wermelt, Jochen Hubertus

**Affiliations:** ^1^Department of Pediatrics and Neonatology, Dr. von Hauner Children’s Hospital, Ludwig-Maximilians-University Munich, Munich, Germany; ^2^Department of Internal Medicine, University Hospital Augsburg, Augsburg, Germany; ^3^Department of Anesthesiology, Ludwig-Maximilians-University Munich, Munich, Germany; ^4^Department of Anesthesiology and Pediatric Anesthesiology, Bürgerhospital Frankfurt am Main, Frankfurt am Main, Germany; ^5^Department of Pediatric Surgery, Ruhr-University Bochum, Bochum, Germany

**Keywords:** neonates, prematurity, ELBW, anesthesia, inguinal hernia repair

## Abstract

**Introduction:**

The purpose of this study was to determine the earliest timing of inguinal hernia repair under general anesthesia with minimized risk for respiratory complications during postoperative course.

**Methods:**

We performed a monocentric analysis of patient records of premature and full-term infants undergoing inguinal hernia repair between 2009 and 2016. In addition to demographic and medical parameters, preexisting conditions and the perioperative course were recorded.

**Results:**

The study included 499 infants (preterm *n* = 285; full term *n* = 214). The number of subsequently ventilated patients was particularly high among preterm infants with bronchopulmonary dysplasia, up to 45.3% (*p* < 0.001). Less than 10% of subsequent ventilation occurred in preterm infants after 45 weeks of postmenstrual age at the time of surgery or in patients with a body weight of more than 4,100 g. Preterm infants with a bronchopulmonary dysplasia had an increased risk of apneas (*p* < 0.05). Only 10% of the preterm babies with postoperative apneas weighed more than 3,600 g at the time of surgery or were older than 44 weeks of postmenstrual age.

**Conclusion:**

Our data indicate that after the 45th week of postmenstrual age and a weight above 4,100 g, the risk for respiratory failure after general anesthesia seems to be significantly decreased in preterm infants.

## Introduction

Infantile inguinal hernia is a common condition, with an incidence in 3–5% of mature infants. Major risk factors are prematurity and male gender. Thus, the incidence of inguinal hernia increases up to 30% in extremely premature infants (< 1,000 g birth weight) (1–3).

Since spontaneous closure is not expected, the diagnosis indicates surgery, usually performed as an elective procedure. An unreducible incarceration is an emergency that must be operated on within 6 h. An urgent indication for surgery arises in the case of poorly reducible inguinal hernias or a prolapsed ovary. In all other premature and full-term infants, it is recommended that surgical correction be performed before the infant is discharged to avoid incarceration at home (4).

This surgical point of view is opposed by the neonatological and anesthesiological points of view. Being exposed to general anesthesia (GA) can create a critical condition for any patient, but special aspects apply to premature and full-term neonates regarding negative outcomes from anesthesia. Among others, Sall showed in an animal model that neural cell death occurs under anesthesia (5). However, McCann et al. showed in a large-scale, prospective study that, after 5 years, neurological outcomes did not appear to be affected (6). Furthermore, a correlation between behavioral abnormalities, developmental delays, and speech disorders after GA before 3 years of age was documented (7). However, other organ systems are also negatively affected. For example, almost 90% of infants present with anuria during anesthesia for laparoscopy (8). Several studies have shown that, especially in preterm infants, autoregulation of cerebral perfusion responds inadequately to fluctuations in pCO_2_ pressure (9–11). Another finding of the GAS study was that infants showed mild to moderate hypotonia intraoperatively (12). Finally, invasive ventilation during anesthesia has a negative impact on existing bronchopulmonary dysplasia (BPD). BPD is a severe lung disease that can progress in preterm infants during prolonged invasive ventilation (13).

For optimal treatment of this vulnerable patient group, the individual aspects for and against an early hernia repair, as well as the choice of anesthetic procedure, must be weighed carefully against each other. This is especially relevant, because less invasive methods using ultrasound-guided regional anesthesia offer the possibility of avoiding GA. On the other hand, the increasing popularity of laparoscopic herniorrhaphy requires GA and invasive ventilation.

Our hypothesis is that prematurity significantly increases the risk for apneas and respiratory failure in the postoperative course. The aim of this study was to specify the perioperative risks of GA in premature infants on the basis of a large patient population. Another aim was to determine the earliest timing of inguinal hernia repair under GA with minimized risk for respiratory complications in the postoperative course.

## Materials and methods

Monocentric analysis of patient records of premature and full-term infants treated for inguinal hernia between 2009 and 2016 was performed. The study was conducted in accordance with the ethical standards of the institutional research committee (Ethics committee of the medical faculty of the Ludwig-Maximilians-Universität München, Ref no. 17–373). Inclusion criteria were open surgical inguinal herniotomy before completing the first year of life and surgery under GA. Exclusion criteria were surgery beyond the first year of life, laparoscopic inguinal herniotomy, and surgery under regional anesthesia. In addition to demographic data such as sex, birth weight, gestational age, and age at the time of surgery, medical parameters regarding preexisting conditions and the perioperative course were recorded.

Preterm infants are defined as having a gestational age < 37 weeks. Infants with a birth weight < 1,500 g are categorized as very low birth weight (VLBW), and infants with a birth weight < 1,000 g are categorized as extremely low birth weight (ELBW). Preterm infants with birth weights above 1,500 g were categorized as “other.” The “duration of anesthesia” comprises the entire anesthesia time from intubation to extubation in the operating room or until transfer to the intensive care unit. Subsequent ventilation time on the ward is recorded separately. Patients were classified as subsequently ventilated when they were extubated in the intensive care unit, and prolonged ventilation was defined as longer than 6 h. Apneas were defined as cessations of breathing beyond 20 s. The indication for red blood cell (RBC) transfusion was based on the recommendations for premature and newborn infants in the *Cross-Sectional Guidelines for Therapy with Blood Components and Plasma Derivatives of the German Medical Association (2014)* (14).

In this study, the definition and classification of BPD severity was based on the 2001 NIH consensus definition (15). Surgery was classified as urgent or an emergency if there was an indication to perform surgery within the next 6 h because of an inguinal hernia that could not be reduced, defined as N0–N2 by *The German Perioperative Procedural Time Glossary* (16).

Statistical analysis and figure design were performed using IBM SPSS Statistics^®^ (version 25.0.0.1) and GraphPad Prism^®^ (version 8.4.3). Medians, quartiles, and frequencies were calculated. The Mann–Whitney test, was used to compare the medians of the independent samples. The significance level was set at *p* < 0.05. To calculate the probability of a group for a given event, absolute risk (AR) was calculated. Relative risk (RR) was calculated to compare the risk of the two groups. Spearman’s rank correlation was used to calculate linear relationships between two variables.

## Results

**FIGURE 1:**
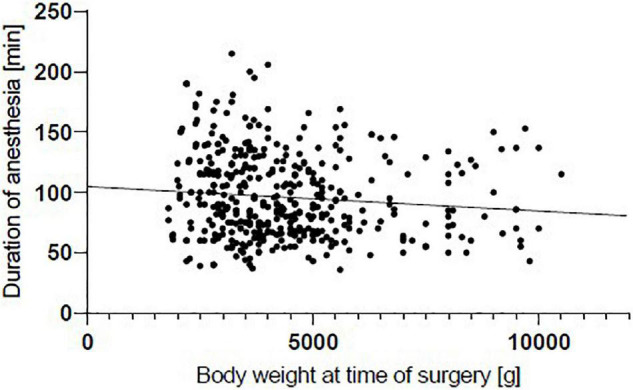
Correlation between duration of anesthesia and weight at time of surgery. Negative correlation shows that duration of anesthesia decreases with increasing body weight (*p* < 0.05; *r* = –0.135).

We identified 499 premature and mature infants who underwent inguinal hernia repair between 2009 and 2016 (shown in Table 1). 413 (82.8%) were male, and 285 (57.1%) were preterm infants, of whom 98 (34.4%) were ELBW. Preterm infants were born at a median of 30.9 gestational weeks. BPD was seen exclusively in the preterm group and here mainly in ELBW. This subgroup represented 67.3% of the subjects. There was only one (0.8%) patient with BPD in preterm infants with a birth weight above 1,500 g. Preterm infants were significantly lighter than full-term infants at the time of surgery (3,400 g; vs. 4,900 g; *p* < 0.0001), although they were operated on significantly later in relation to chronological age (79 days vs. 56 days; *p* < 0.0001) (shown in Table 2). At median, preterm infants were almost three times as likely to have bilateral inguinal hernias than full-term infants (29.5% vs. 10.5%; *p* < 0.0001). In preterm infants, ELBW showed a particularly high proportion of bilateral inguinal hernias. Thus, 43.9% of all ELBW had bilateral involvement, whereas this proportion dropped to 18.2% in preterm infants above 1,500 g birth weight. Similarly, preterm infants had a significantly longer duration of anesthesia than full-term infants (100 min vs. 80 min; *p* < 0.0001). The time before, during and after the surgical intervention was considered as the duration of the anesthesia. While the duration of anesthesia differs by 12 min for unilateral inguinal hernias, the difference for bilateral inguinal hernias is 16 min when comparing preterm and full-term infants. Overall, there was a correlation between lower weight at the time of surgery and a longer time under anesthesia (*p* < 0.05; *r* = –0.135) (shown in Figure 1). The frequency of urgent surgery because of incarcerated inguinal hernia was the same in both groups, approximately 18%.

**TABLE 1:** Patient characteristics.

	Preterm (*n* = 285)	Full term (*n* = 214)	
**Sex**			
Male	243 (85.3%)	170 (79.4%)	
Female	42 (14.7%)	44 (20.6%)	
Birth weight, median (p25–p75), [g]	1,310 (830–2,005)	3,100 (2,785–3,500)	
Gestational age, median (p25–p75), [weeks]	30.9 (27.3–34.4)		
BPD	75 (26.3%)	0	
**Bilateral hernia**			
All preterm	84 (29.5%)	22 (10.5%)	*p* < 0.0001, RR = 2.9, OR = 3.6
ELBW (*n* = 98)	43 (43.9%)		*p* < 0.0001, RR = 4.3, OR = 6.8
VLBW (*n* = 63)	19 (30.2%)		*p* < 0.001, RR = 2.9, OR = 3.8
Other (*n* = 121)	22 (18.2%)		*p* < 0.05, RR = 1.8, OR = 1.9

p25, 25th percentile; p75, 75th percentile; BPD, bronchopulmonary dysplasia; RR, relative risk; OR, odds ratio; ELBW, extremely low birth weight; VLBW, very low birth weight; Other, preterm infants > 1,500 g birth weight.

**TABLE 2:** Perioperative characteristics of the cohort.

	Preterm (*n* = 285)	Full term (*n* = 214)	
Chronological age at time of surgery, median (p25–p75), [d]	79 (56–105)	56 (40–97)	*p* < 0.0001
Weight at time of surgery, median (p25–p75), [g]	3,400 (2,785–4,310)	4,900 (4,000–6,185)	*p* < 0.0001
**Duration of anesthesia, median (p25–p75), [min]**			
Unilateral herniorrhaphy	88 (73–115)	76 (63–107)	*p* < 0.001
Bilateral herniorrhaphy	130 (107–142.5)	114 (82–147.5)	n.s.
Combined	100 (75–126)	80 (64–110)	*p* < 0.0001
Emergency operation indication	53 (18.6%)	39 (18.2%)	n.s.
Hemoglobin pre-op, median (p25–p75), [g/dl]	10.7 (9.8–11.7)	11.2 (10,0–12.0)	n.s.
Hct pre-op, median (p25–p75), [%]	31 (28–34)	32 (29.75–35)	n.s.
**Pre-op RBC transfusion**			
All preterm	14 (4.9%)	1 (0.5%)	*p* < 0.01, RR = 10.5, OR = 11.0
ELBW (*n* = 98)	10 (10.2%)		*p* < 0.0001, RR = 21.8, OR = 24.2
VLBW (*n* = 63)	2 (3.2%)		n.s.
Other (*n* = 121)	2 (1.7%)		n.s.
**Post-op RBC transfusion**			
All preterm	16 (5.6%)	0	*p* < 0.0001
ELBW (*n* = 98)	6 (6.1%)		*p* < 0.001
VLBW (*n* = 63)	4 (6.3%)		*p* < 0.01
Other (*n* = 121)	6 (5.0%)		*p* < 0.01
Minimal pCO_2_ intra-op, median (p25–p75), [mmHg]	33 (28–36)	35 (33–38)	*p* < 0.0001
Minimal MAP intra-op, median (p25–p75), [mmHg]	38 (30–45)	45 (38–52)	*p* < 0.0001
Subsequent ventilation	80 (28.1%)	4 (1.9%)	*p* < 0.0001, RR = 15.0, OR = 20.5
In preterm infants with BPD	34 (45.3%)		*p* < 0.0001, RR = 24.3, OR = 43.5
In preterm infants without BPD	46 (22.0%)		*p* < 0.0001, RR = 7.9, OR = 9.1
Prolonged ventilation > 6 h	22 (7.7%)	1 (0.5%)	*p* < 0.0001, RR = 16.5, OR = 17.8
Post-op apneas	35 (12.3%)	0	*p* < 0.0001

p25, 25th percentile; p75, 75th percentile; n.s, not significant; RR, relative risk; OR, odds ratio; Hct, hematocrit; RBC, red blood cell; ELBW, extremely low birth weight; VLBW, very low birth weight; Other, preterm infants > 1,500 g birth weight; pCO_2_, carbon dioxide partial pressure; MAP, mean arterial pressure.

### Red blood cell transfusion

The preterm and full-term infants did not differ in terms of hematocrit or hemoglobin level. A total of 15 children required RBC transfusions for preoperative preparation. This proportion was particularly high in the ELBW group. Here, a total of 10 (10.2%) patients received an RBC transfusion preoperatively. Postoperatively, 16 patients required an RBC transfusion, and they were all preterm infants. However, the subgroups did not differ from each other.

### Anesthetic management

A more pronounced hypocapnia occurred more frequently in preterm infants. The median of the minimal pCO_2_-values was lower in the group of preterm infants than in full-term patients (33 mmHg vs. 35 mmHg; *p* < 0.0001). Preterm infants also had significantly lower mean arterial pressures during anesthesia (38 mmHg vs. 45 mmHg; *p* < 0.0001).

### Postoperative course

**FIGURE 2:**
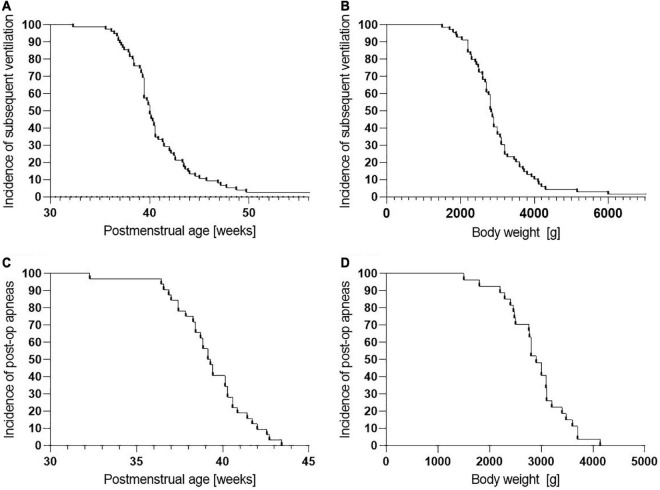
Cumulative frequency of subsequent ventilation and postoperative apneas depending on gestational age and body weight at time of surgery. **(A)** Subsequent ventilation depending on postmenstrual age (PMA) at time of surgery. Less than 10% of delayed extubations occurred after 45 weeks of PMA. **(B)** Subsequent ventilation depending on body weight at time of surgery. Less than 10% of delayed extubations occurred with a body weight of more than 4,100 g. **(C)** Post-op apneas depending on PMA at time of surgery. Less than 10% of post-op apneas occurred after 42 weeks of PMA. No post-op apneas occurred after 44 weeks of PMA. **(D)** Post-op apneas depending on body weight at time of surgery. Less than 10% of post-op apneas occurred with a body weight of more than 3,600 g. No post-op apneas occurred beyond 4,100 g.

Eighty (28.1%) of the preterm infants required subsequent mechanical ventilation, while only 4 (1.9%) of the full-term infants required subsequent mechanical ventilation (*p* < 0.001). The number of subsequently ventilated patients was particularly high among those with BPD. Here, the proportion increased to 45.3% (*p* < 0.0001). Overall, there was a 22.5-fold risk of subsequent ventilation in preterm infants with BPD compared to full-term infants. In terms of postmenstrual age (PMA) at the time of inguinal hernia repair, it was shown that less than 10% of subsequent ventilation occurred after 45 weeks of PMA in preterm infants (shown in Figure 2A). In terms of weight at surgery, less than 10% of subsequent ventilation occurred with a body weight of more than 4,100 g (shown in Figure 2B). Furthermore, ventilation prolonged beyond 6 h was still necessary in 22 preterm infants and only 1 full-term infant (7.7% vs. 0.5%; *p* < 0.001).

Only preterm infants showed postoperative apneas, with the highest incidence in the ELBW group. Analogous to the subsequent ventilation, infants with a BPD had a significantly increased risk for apneas (*p* < 0.05; RR = 2.6; OR = 3.3). Less than 10% of the preterm infants with postoperative apneas weighed more than 3,600 g at the time of surgery, respectively, were older than 44 weeks of PMA (shown in Figures 2C,D).

## Discussion

Inguinal hernia is a common condition, especially in premature infants, and it occurs in a vulnerable phase of life. In most hospitals, the herniotomy is performed under GA, which can be challenging for this age group. However, the question arises whether the disadvantages of early inguinal hernia repair under GA outweigh the advantages and how risks could be minimized in a standardized manner. One way was to perform the operations under awake caudals. However, this type of anesthesia has not yet been established everywhere. In addition, laparoscopic herniorrhaphy is becoming increasingly standard, so that even in hospitals that had introduced awake caudals, children are now operated on under GA again.

For analysis, we divided our cohort into preterm and mature infants. In this cohort, inguinal hernia repair had to be performed urgently or even as an emergency in approximately 18% in both groups. This corresponds to findings reported in the literature for infants. Rates of incarceration between 15 and 35% have been reported (17–19). This rather high rate of urgent or emergency herniotomies justifies performing the hernia repair in case of prolonged postnatal hospitalization even before discharge from the hospital to avoid incarceration in the family environment.

However, we were able to show that preterm infants had a significantly lower weight at the time of surgery, although they were significantly older in terms of chronological age. Regarding the corrected age, preterm infants underwent surgery earlier than term infants. These results are consistent with those of Massoud et al. (20). Moreover, preterm infants not only underwent surgery earlier in life, the proportion of bilateral inguinal hernias was 29.5%, about three times as high as in full-term infants. ELBW has a particularly high proportion at 43.9%, similar to that reported in the literature (2, 21, 22). This is certainly a major factor why the periods under anesthesia are particularly long in preterm infants (100 min vs. 80 min for unilateral and bilateral hernia repair combined, *p* < 0.0001). Unilateral inguinal hernia repair also lasts significantly longer in preterm infants (88 min vs. 76 min, *p* < 0.001). Due to the small number of term infants with bilateral hernia repair, no level of significance was reached, although the median time under anesthesia was 16 min longer in preterm infants. According to the hospital standard, premature babies were operated by experienced surgeons. Younger surgeons, on the other hand, are trained on older patients before they operate on inguinal hernias in full-term infants. When weight at the time of surgery is compared with the duration under anesthesia, an inverse correlation is obtained. The duration of anesthesia decreases as body weight increases (*p* < 0.05, *r* = –0.135) (shown in Figure 1). This shows that among all infants, the most vulnerable patient group has the longest exposure to anesthesia.

Thus, this longer anesthesia time is encountered in a group with a high rate of preterm pulmonary disease. BPD is a common condition in preterm infants (13, 15). In our study, only preterm infants were affected by BPD (26.3%). This percentage increases to 67.3% in ELBW. Besides lung immaturity, invasive ventilation is the main factor for the development of BPD. We demonstrated that not only did preterm infants have a longer anesthesia time, but that the rate of subsequent ventilation was particularly high for preterm infants with BPD. Nearly half of the infants with BPD required subsequent ventilation (45.3%). Among preterm infants without BPD, the proportion drops to 22%. Less than 2% of full-term infants required subsequent ventilation. The result that BPD is a risk factor for subsequent ventilation is consistent with a significant study by Lamoshi et al. (23). Because of the larger cohort in this study, it is also possible to quantify the relative risk (RR) of subsequent ventilation for different subgroups (Table 2).

A similar picture emerges for apneas occurring postoperatively. These did not occur in full-term infants but occurred in 23.5% of the ELBW infants.

While these findings strongly suggest that respiratory complications after inguinal hernia repair under GA particularly affect preterm infants, ELBW infants with BPD are affected even more. If we consider the decrease of risks for postoperative respiratory failure or the occurrence of postoperative apnea, our study shows that less than 10% of these complications occur beyond a PMA of 45 weeks at surgery and/or a weight of more than 4,100 g (shown in Figure 2). This is consistent with the findings of Malviya et al. (24), for the first time supplemented by a weight limit above which such complications are rare.

While McCann et al. could show in the GAS study that GA in infants seems not to be critical with regard to neurological development (6), our data argue for a clearly differentiated approach. We showed that, especially in preterm infants and particularly in ELBW, there is a significant risk of respiratory complications in the postoperative course. This could be avoided by using awake caudals, the effectiveness of which was shown by Davidson et al. and others (25). However, laparoscopic herniorrhaphy is increasingly becoming the standard of care in children of all ages, as postulated by Zani et al. (26). Our data suggest that GA should be delayed in preterm infants until the 45th week of PMA and/or they attain a weight greater than 4,100 g. If surgery is indicated before these values are reached, awake caudals should still be considered.

Limitations of this study are mainly its retrospective approach. However, because of close perioperative monitoring, the data could be reconstructed properly from medical records. The relatively high number of patients compared to other studies allowed statistical analysis of subgroups.

## Conclusion

In summary, the relatively high incarceration rate of 15–35% justifies an early hernia repair before patients are discharged home. On the other hand, our data demonstrate that preterm infants, especially ELBW infants or patients with BPD, are at high risk of postoperative respiratory complications.

We emphasize that GA should be critically discussed for this vulnerable patient group, particularly since several studies have shown that postoperative respiratory complications do not occur to the same extent after RA. After the 45th week of PMA and in patients weighing more than 4,100 g, GA seems to be less problematic.

## Data availability statement

The raw data supporting the conclusions of this article will be made available by the authors, without undue reservation.

## Ethics statement

The studies involving human participants were reviewed and approved by Ethikkommission der Medizinischen Fakultät der LMU Muenchen Pettenkoferstr. 8 D-80336 Muenchen Germany. Written informed consent from the participants’ legal guardian/next of kin was not required to participate in this study in accordance with the national legislation and the institutional requirements.

## Author contributions

SS conceptualized and designed the study, supervised data collection and analysis, drafted, and finalized the manuscript. PM carried out all data collection and analysis and reviewed the manuscript. MK and JW conceptualized the study, reviewed the analysis, and the manuscript. JH conceptualized and designed the study, supervised the analysis and critically reviewed and revised the manuscript. All authors approved the final manuscript as submitted and agree to be accountable for all aspects of the work.

## Abbreviations

AR: Absolute risk
BPD: Bronchopulmonary dysplasia
ELBW: Extremely low birth weight
GA: General anesthesia
OR: Odds ratio
p25: 25th percentile
p75: 75th percentile
PMA: Postmenstrual age
RBC: Red blood cell
RR: Relative risk
SD: Standard deviation
VLBW: Very low birth weight.
